# Genome-wide identification of the class III *POD* gene family and their expression profiling in grapevine (*Vitis vinifera* L)

**DOI:** 10.1186/s12864-020-06828-z

**Published:** 2020-06-29

**Authors:** Huilin Xiao, Chaoping Wang, Nadeem Khan, Mengxia Chen, Weihong Fu, Le Guan, Xiangpeng Leng

**Affiliations:** 1 College of Horticulture, Nanjing Agricultural University, Nanjing, 210095 P. R. China; 2 Yantai Academy of Agricultural Sciences, Yantai, 264000 P. R. China; 3 Ottawa Research and Development Center, Agriculture and Agri-Food Canada, Ottawa, Ontario K1A 0C6 Canada; 4 Department of Biology, University of Ottawa, 30 Marie Curie, Ottawa, ON K1N 6N5 Canada; 5 College of Horticulture, Qingdao Agricultural University, Qingdao, 266109 P. R. China

**Keywords:** Grapevine, Genome-wide analysis, *POD* genes family, Collinearity and expression analysis

## Abstract

**Background:**

The class III peroxidases (PODs) are involved in a broad range of physiological activities, such as the formation of lignin, cell wall components, defense against pathogenicity or herbivore, and abiotic stress tolerance. The POD family members have been well-studied and characterized by bioinformatics analysis in several plant species, but no previous genome-wide analysis has been carried out of this gene family in grapevine to date.

**Results:**

We comprehensively identified 47 PODs in the grapevine genome and are further classified into 7 subgroups based on their phylogenetic analysis. Results of motif composition and gene structure organization analysis revealed that PODs in the same subgroup shared similar conjunction while the protein sequences were highly conserved. Intriguingly, the integrated analysis of chromosomal mapping and gene collinearity analysis proposed that both dispersed and tandem duplication events contributed to the expansion of PODs in grapevine. Also, the gene duplication analysis suggested that most of the genes (20) were dispersed followed by (15) tandem, (9) segmental or whole-genome duplication, and (3) proximal, respectively. The evolutionary analysis of PODs, such as *Ka/Ks* ratio of the 15 duplicated gene pairs were less than 1.00, indicated that most of the gene pairs exhibiting purifying selection and 7 pairs underwent positive selection with value greater than 1.00. The *Gene Ontology Enrichment (GO), Kyoto Encyclopedia of Genes Genomics* (KEGG) analysis, and cis-elements prediction also revealed the positive functions of PODs in plant growth and developmental activities, and response to stress stimuli. Further, based on the publically available RNA-sequence data, the expression patterns of PODs in tissue-specific response during several developmental stages revealed diverged expression patterns. Subsequently, 30 genes were selected for RT-PCR validation in response to (NaCl, drought, and ABA), which showed their critical role in grapevine.

**Conclusions:**

In conclusion, we predict that these results will lead to novel insights regarding genetic improvement of grapevine.

## Backgroud

Peroxidases (EC 1.11.1.X) is a group of well-known large multi-gene family and that are broadly dispersed in living organisms. They catalyze oxidative reactions using hydrogen peroxide (H_2_O_2_) as the electron acceptor in their active center with a metal. Based on their structure variations, the peroxidase (PODs) can be characterized into two main groups such as either heme PODs and non-heme PODs [1]. Meanwhile, the heme PODs can be ordered into two more sub-families like animal PODs and non-animal PODs [2]. The non-animal superfamily contains three major sub-distinct classes namely, class I, II, and III [3]. The class III peroxidases (EC 1.11.1.7) are abbreviated in various ways in previous studies (POX, POD, Px, PER, and Prx) and act as plant-specific oxidoreductases [3, 4]. In this study, we will use the abbreviation for class III peroxidase as POD. The class III plant peroxidase (POD) plant is a plant-specific oxidoreductas, which is one of the many types of peroxidases that are widely distributed in animals, plants and microorganisms [3] . In plants growth, they are also known for their dual role in both cell wall hardening as well softening [5]. The PODs are involved in in various processes (e.g. lignification, plant defense, development, germination) and their mechanisms of action (substrate oxidation, regulation of reactive oxygen species and the formation of radicals), focusing specifically on lignification [6–10].

In recent times, due to the results of transcriptomic data, a large number of PODs have been accompanying numerous biological processes [11–13]. However, the direct role of this multi-gene family is still elusive and only a few studies have demonstrated their functional role [5, 8, 14, 15]. For example, *Arabidopsis thaliana* and *Populus trichocarpa* PODs (*AtPrx72* and *PtrPO21*), play a significant role in lignification of the leaves [13, 16]. The overexpression of the *POD* genes in *A. thaliana* (*AtPrx22, AtPrx39, and AtPrx69*) improve cold tolerance [17]. Moreover, the cotton *GhPOX1* has been studied for a higher production level of reactive oxygen species [18]. Several, *POD* genes in roots of *Zea mays* are known to regulate by methyl jasmonate, salicylic acid and pathogen elicitors [19]. Taken together, based on these results, the PODs play an important role in biological, physiological, and in response to stress stimuli, therefore, its comprehensive analysis is necessary to further explore its role in plant growth and development.

In addition, the POD family members have been well-studied and characterized by bioinformatics analysis in several plant species including, 73 PODs in *Arabidopsis thaliana* [20], 138 in *Oryza sativa* [21], 93 in *Populus trichocarpa* [22], 102 in *Medicago sativa* [23], 119 in *Zea mays* [24]*,* 94 in *Pyrus bretschneideri* [25]. Nevertheless, to date, no previous genome-wide analysis has been carried out of this gene family in grapevine. While, a large number of genes in this family suggested their functional diversity among each individual proteins [12]. Grapevine (*Vitis vinifera* L) is one of the widely popular and important fruit crops in the world [24]. A common goal of current plant genomics research is to create an expandable platform for global classification and analysis of plant gene family. Hence, it’s necessary to provide a foundation for future research. In the meantime, the availability of the grapevine genome (Version 2.1) facilitate the research in grapevine momentously for its genetic studies by improvement in the quality of berry.

In the present study, we performed a wide-ranging bioinformatics analysis of *POD* gene family and verified their role against various stress responses (i-e., NaCl, drought, and ABA) in grapevine. In total, 47 genes were identified for the first time in the grapevine genome and were systematically analyzed by genome-wide approaches. Thus, the study including, physicochemical properties, phylogenetic relationships, chromosomal mapping, collinear correlation, gene duplication events, rate of substitution rates, motif composition and gene structure, promoter sequence analysis, GO and KEGG enrichment analysis, and expression profiling using RNA-seq data and RT-PCR analysis in response to salt, drought and Abscisic acid (ABA). In general, the results of our study will undoubtedly be helpful for future research on fruits crop species and pay the base for functional characterization of the *PODs* gene family.

## Results

### Characterization of POD gene family in grapevine

In this study, a total of 47 *POD* genes were identified from the grapevine genome and for simplicity, we denominated as *VvPOD1-VvPOD47* based on their orthologous position with *Arabidopsis thaliana*. We also studied some useful information of PODs including, the protein identifier, chromosomal localization, coding sequence (CDS) length (bp), and various physicochemical properties such as, protein length (aa), molecular weight (MW) kDa, isoelectric point (PIs), and grand average of hydropathicity (GRAVY). While, the gene duplication types (i.e., dispersed, tandem, proximal, and segmental or whole-genome duplication) and subcellular localization analysis were also briefly studied for each of POD proteins (Supplementary Table S1). In brief, the CDS length varies from 801 bp (*VvPOD35*) to 2188 bp (*VvPOD12*) with an average of 1009.021 bp. Similarly, the protein length varies from 266 aa (*VvPOD35*) to 705 aa (*VvPOD12*) with an average of 335.34 aa, respectively. Also, the MW ranged from 28.50 kDa (*VvPOD35*) to 76.17 kDa (*VvPOD12*) with mean MW of 36.48 kDa, the PIs varies from 4.16 (*VvPOD47*) to 9.56 (*VvPG13*), respectively. The results of GRAVY ranged from − 0.37 (*VvPOD10*) to 0.03 (*VvPOD25*). Intriguingly, the variability was observed in most of the genes for GRAVY, indicating mostly hydrophilic properties and only a few of them (*VvPOD46*, *VvPOD43*, *VvPOD31*, and *VvPOD25*) are hydrophobic in nature by showing positive values. Additionally, the gene duplication analysis intimated that most of the genes (20) were dispersed followed by tandem (15), segmental or whole-genome duplication (9), and proximal (3), respectively.

### Phylogenetic relationships, gene structure organization of POD gene family in grapevine

**Fig. 1:**
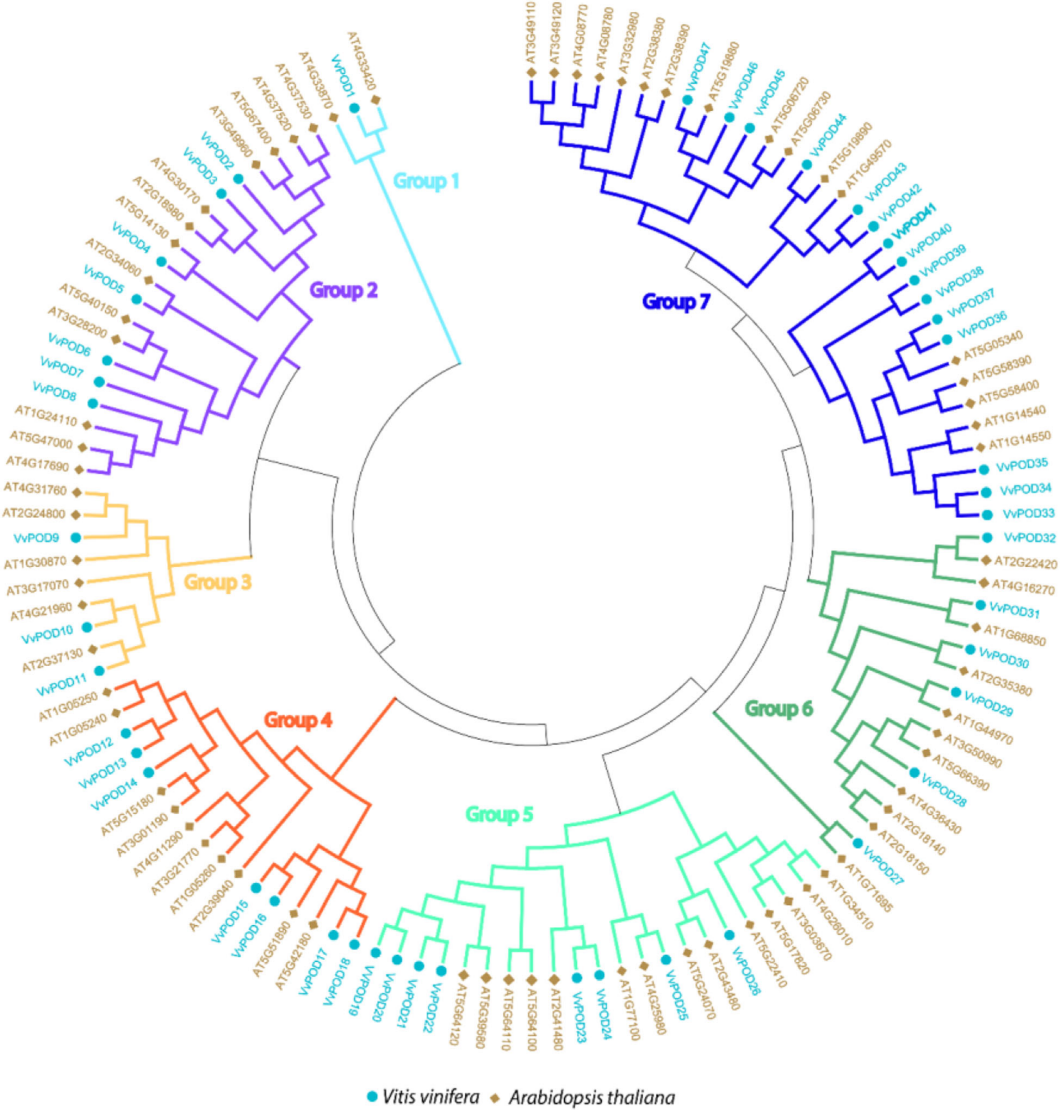
Phylogenetic relationship of *POD* genes between grapevine and *Arabidopsis*. The phylogenetic tree was constructed by MEGA 7.0 using the Maximum Likelihood Method (1000 bootstrap)

To investigate the evolutionary relationships, we used the 47 *POD* gene grapevine and 73 *Arabidopsis thaliana* to construct a maximum likelihood approach tree by using MEGA 7.0. The phylogenetic tree reveals that PODs can be further subcategorized into 7 subgroups (Fig. 1). The results exhibited that there is an uneven distribution of *VvPOD* genes compared with *AtPODs*. For instance, we observed that subgroup 7 contains the most number of genes (15 and 17) as compared to other subgroups in grapevine and *Arabidopsis*. The phylogenetic tree also revealed the relatively close genetic relationships with *Arabidopsis*.

**Fig. 2:**
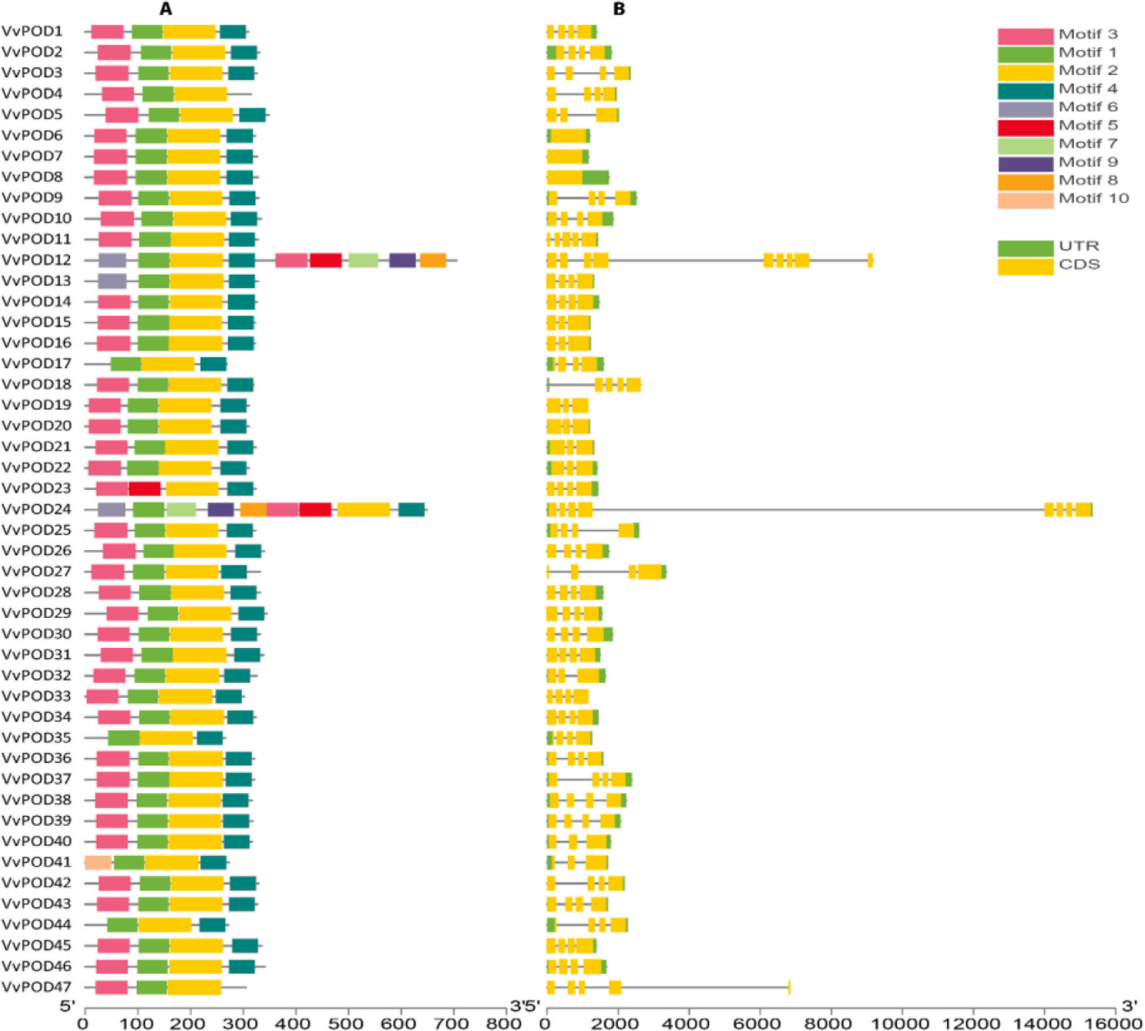
**a** and **b**. **a** Motif composition of POD in grapevine are presented in different color ranging from motif 1–10. **b** The coding sequences (CDS) and untranslated regions (UTR) for PODs in grapevine are represented by yellow and green boxes, respectively. Motif composition and gene structure were visualized by TBtools software. At the bottom of the figure, the relative position is proportionally displayed based on the kilobase scale

The 10 conserved motifs ranging from (motif 1–10) of VvPODs were explored by the MEME program. Markedly, motifs 1–4 were most common among the members of PODs, suggesting unique features among subgroups (Fig. 2a). Also, the LOGOS for these motifs were obtained by MEME, the higher number (100) consensus sequences were observed in motif-2 while a less number (50) were recorded in motif 4, and motifs 8–10, respectively (Supplementary Figure S1). The gene structure organization was analyzed based on CDS and untranslated regions (UTRs) by using TBtools. The result reveals that *VvPOD* members are highly conserved within each other and displayed a similarity among subgroups (Fig. 2 b). Further, these findings indicated the structural diversification among *VvPOD* gene family.

### Chromosomal localization, gene collinearity, and Ka/Ks analysis of POD

**Fig. 3:**
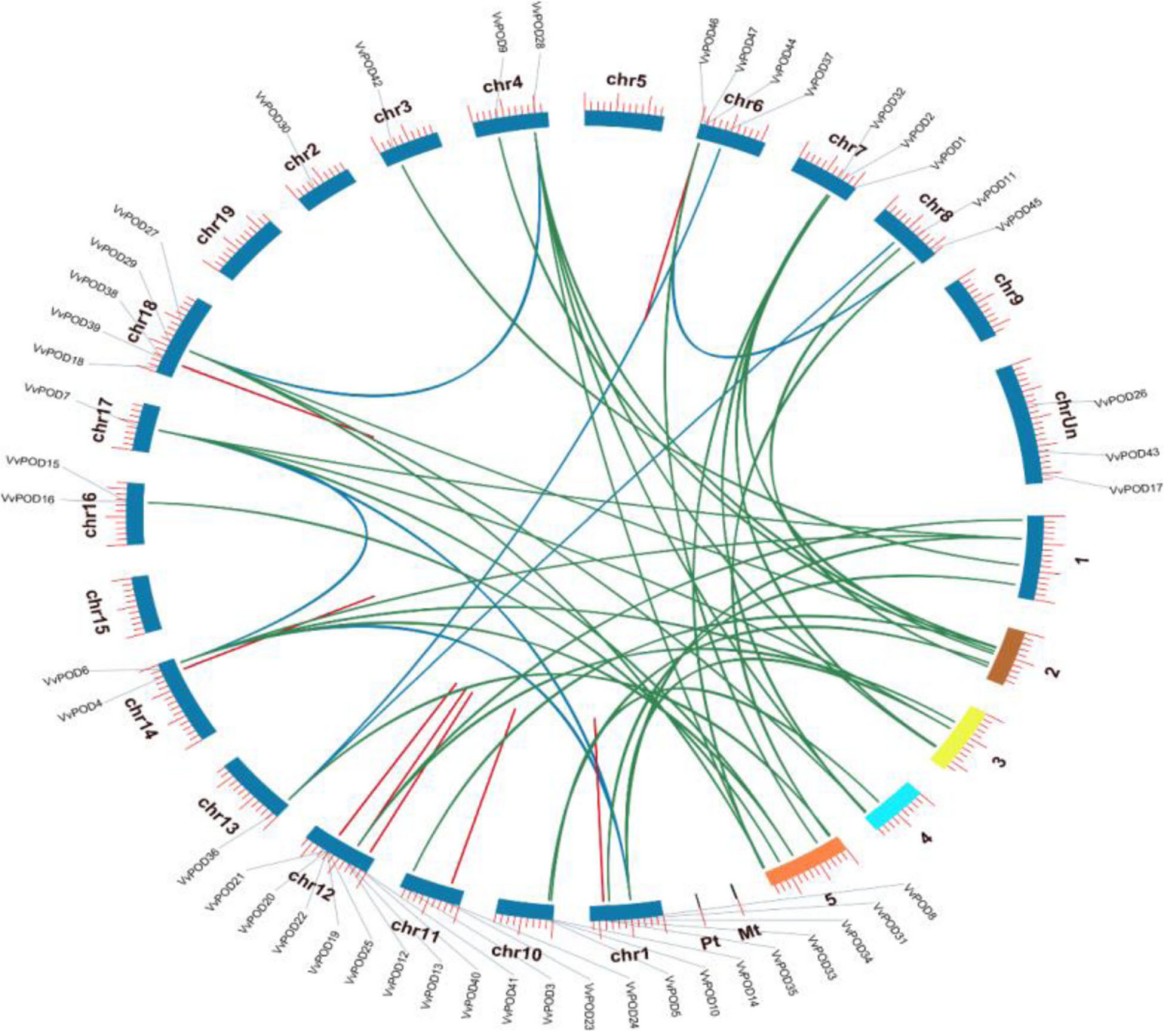
**a** and **b. a** The chromosomal localizations are shown for grapevine (Chr01–19) is blue and for *Arabidopsis* different random colors. **b** The collinear correlation at the center for all the *POD* genes is displayed between grapevines and *Arabidopsis*. The green line indicates the collinear relationship among *VvPODs* and *AtPODs*, blue represent the relation within *VvPOD* and red lines indicates the tandem duplications

To illustrate the chromosomal localization among 47 POD members and the gene collinearity analysis between grapevine and *Arabidopsis* were drawn with the help of TBtools software. The results for PODs chromosomal localization unveiled the irregular distribution patterns ranging from 1 to 9 proteins per chromosome except (chr5, chr9, and chr15) across 19 different chromosomes (i.e., Chr01-Chr19) in the grapevine genome. Also, the number of genes on each chromosome were distinct such as the high number of genes (9) were observed on Chr12, followed by Chr1 and Chr18 each with 5 genes, chr6 has 4 genes, while 3 genes were allocated on the Chr7, Chr10 and unknown chromosome (ChrUn), respectively, as described in Fig. 3. Thus, among POD members high variation patterns were observed in the grapevine genome. Furthermore, the gene collinearity relationships between *V. vinifera* (*VvPOD*) and *Arabidopsis* (*AtPOD*) was also illustrated by using circos plot with the help of TBtools software. As a consequence, high conservation was observed between *VvPOD* and *AtPOD* genes (Fig. 3).

The selection pressure among various types of duplications (i.e., dispersed, tandem, proximal, segmental or WGD), also intended by calculating the rates of synonymous substitution (*Ks*) and non-synonymous substitution (*Ka*). During evolutionary processes, the genes are typically exposed to various types of selection pressure, such as purifying selection (*Ka/Ks <* 1), positive selection (*Ka/Ks* > 1), and neutral selection (*Ka/Ks* = 1) [26]. Among 47 *VvPOD* members, we selected 22 pairs (i.e., 10 pair dispersed, 1 pair proximal, 7 pair tandem, and 4 pair segmental or WGD) as presented in Table 1. Results showed that most of the gene pairs having less than 1.00 *Ka/Ks* ratio suggested purifying selection, thus revealed limited divergence after gene duplications. Though, 7 pairs were observed with higher than 1.00 values, implicating positive selection.

**Table. 1:** The *POD* genes in grapevine with outlier *Ka/Ks* and various types of duplications of the *POD* gene pairs with the detection by the MCScan algorithm (i.e., Dispersed, proximal, tandem, and segmental)

Gene 1	Gene 2	*Ks*	*Ka*	*Ka*/*Ks*	Selection Pressure	Gene Duplications
*VvPOD1*	*VvPOD2*	0.76	0.65	0.86	Purifying Selection	Dispersed
*VvPOD3*	*VvPOD5*	0.69	0.58	0.83	Purifying Selection	Dispersed
*VvPOD9*	*VvPOD10*	0.68	0.63	0.92	Purifying Selection	Dispersed
*VvPOD11*	*VvPOD14*	0.89	0.65	0.73	Purifying Selection	Dispersed
*VvPOD15*	*VvPOD16*	0.04	0.01	0.28	Purifying Selection	Dispersed
*VvPOD17*	*VvPOD18*	1.41	0.11	0.08	Purifying Selection	Dispersed
*VvPOD25*	*VvPOD26*	0.92	0.62	0.68	Purifying Selection	Dispersed
*VvPOD27*	*VvPOD30*	0.56	0.74	1.33	Positive Selection	Dispersed
*VvPOD31*	*VvPOD32*	0.60	0.64	1.07	Positive Selection	Dispersed
*VvPOD42*	*VvPOD43*	0.95	0.36	0.38	Purifying Selection	Dispersed
*VvPOD24*	*VvPOD35*	0.75	0.43	0.58	Purifying Selection	Proximal
*VvPOD4*	*VvPOD12*	1.20	0.45	0.38	Purifying Selection	Tandem
*VvPOD13*	*VvPOD19*	0.50	0.53	1.05	Positive Selection	Tandem
*VvPOD20*	*VvPOD21*	0.18	0.04	0.24	Purifying Selection	Tandem
*VvPOD22*	*VvPOD23*	0.24	0.56	2.35	Positive Selection	Tandem
*VvPOD33*	*VvPOD34*	0.40	0.05	0.13	Purifying Selection	Tandem
*VvPOD38*	*VvPOD36*	0.20	0.09	0.47	Purifying Selection	Tandem
*VvPOD40*	*VvPOD41*	0.07	0.03	0.35	Purifying Selection	Tandem
*VvPOD6*	*VvPOD7*	0.22	0.37	1.68	Positive Selection	WGD or Segmental
*VvPOD8*	*VvPOD28*	0.57	0.67	1.19	Positive Selection	WGD or Segmental
*VvPOD29*	*VvPOD36*	0.48	0.61	1.28	Positive Selection	WGD or Segmental
*VvPOD37*	*VvPOD45*	0.66	0.63	0.96	Purifying Selection	WGD or Segmental

### Gene ontology enrichment (GO), Kyoto encyclopedia of genes genomics (KEGG) and cis-regulatory elements analysis in grapevine

The GO enrichment analysis for *POD* genes was performed to understand their functional regulatory mechanism by using the orthologous pairs of *Arabidopsis thaliana*. The three common subgroups were observed such as molecular functions (MF), cellular component (CC), and biological process (BP). In the MF processes, “oxidoreductase and catalytic activity” (GO:0016491 and GO:0003824), are highly enriched GO terms. Similarly, for CC processes and BP most of the GO terms are responsive to “cell wall, plasmodesma, symplast, cell-cell junction, plant-type cell wall” (GO:0005618, GO:0009506, GO:0055044, GO:0005911, and GO:0009505), and “response to toxic substance, cellular response to stimulus, oxidation-reduction process, metabolic and cellular process” (GO:0009636, GO:0051716, GO:0055114, GO:0008152, and GO:0009987), and are briefly summarized in Supplementary Table S2. As results, the GO terms for MF, CC, and BP, suggested the crucial role of PODs in various activities of grapevine.

Additionally, the KEGG enrichment analysis indicated the three major pathways among PODs in grapevine such as “Biosynthesis of other secondary metabolites, phenylpropanoid biosynthesis, and metabolism” (Supplementary Table S3).

Moreover, the cis-acting elements in the promoter region of POD members were performed by using the PlantCARE database. In brief, most of the genes were largely participating in light regulation with key regulatory elements (GT1-motif, G-Box, GATA-motif, and AE-Box), followed by hormones (CGTCA-motif, TGACG-motif, ABRE, and GARE-motif), stress and other regulatory factors (LTR, ARE, CCAAT-Box, CAT-BOX, o2-site,), and circadian, respectively. Thus, we observed the diversified role of POD members and their indirect involvement in several biotic-abiotic/hormone signaling processes (Supplementary Table S4).

### Expression profiling of POD genes in different organs and developmental stages in grapevine

**Fig. 4:**
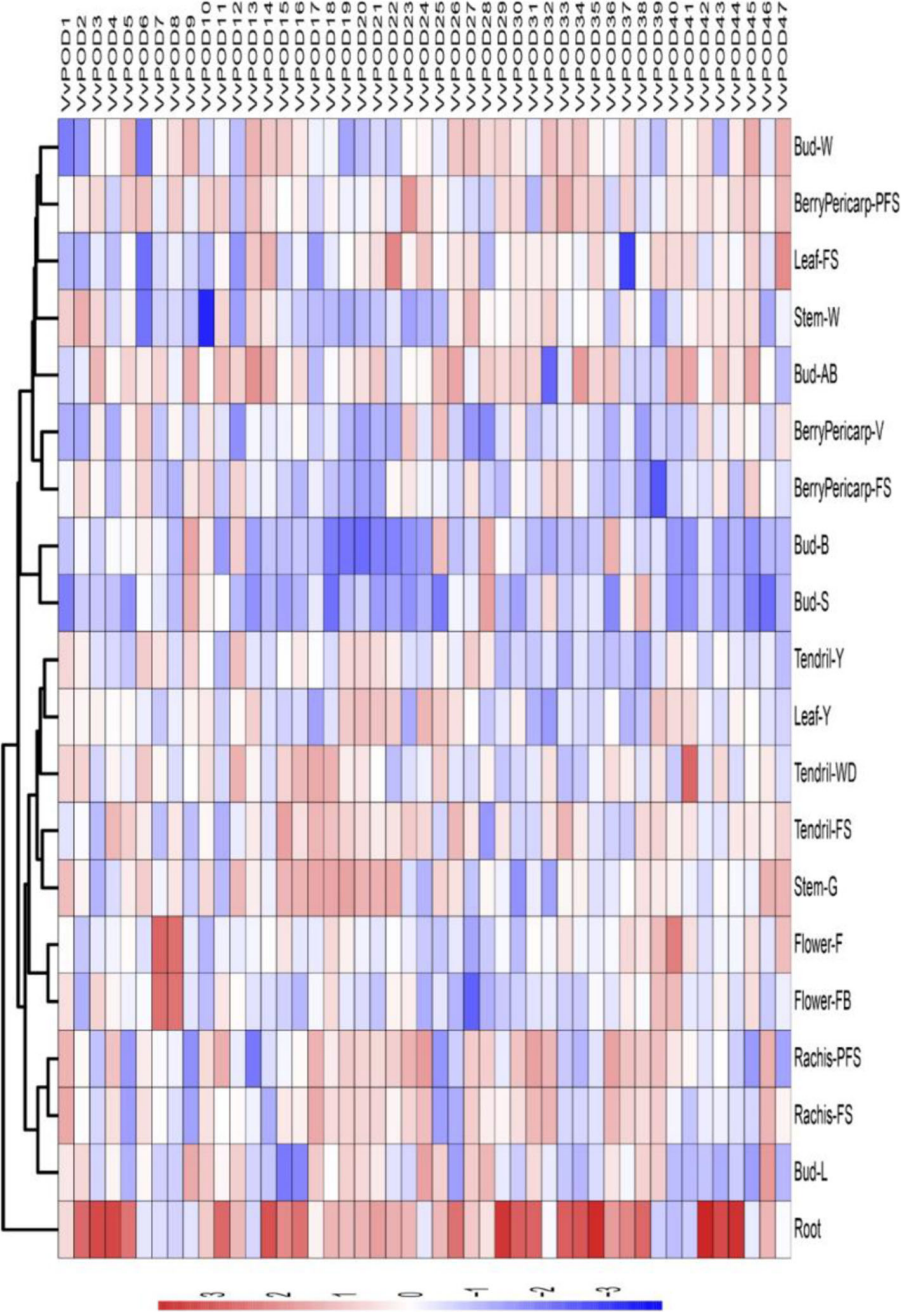
Expression profiles of the 47 *POD* genes in grapevine, including different organs, tissues, and developmental stages. Data were normalized based on the mean expression value of each gene in all tissues analyzed. BerryPericarp-FS: berry pericarp fruit set; BerryPericarp-PFS: berry pericarp post-fruit set; BerryPericarp-V: Bud-S: bud swell; Bud-B: bud burst (green tip); Bud-AB: bud after-burst (rosette of leaf tips visible); Bud-L: latent bud; Bud-W: winter bud; Flower-FB: flowering begins (10% caps off); Flower-F: flowering (50% caps off); Leaf-Y: young leaf (pool of leaves from shoot of 5 leaves); Leaf-FS: mature leaf (pool of leaves from shoot at fruit set); Rachis-FS: rachis fruit set; Rachis-PFS: rachis post fruit set; Stem-G: green stem; Stem-W: woody stem; Tendril-Y: young tendril (pool of tendrils from shoot of 7 leaves); Tendril-WD: well developed tendril (pool of tendrils from shoot of 12 leaves); Tendril-FS: mature tendril (pool of tendrils at fruit set)

The expression profiling of all 47 *POD*s in grapevine derived from 19 tissues and organs during their developmental stages were investigated in the present srudy. The RNA-seq data were retrieved from NCBI database (GSE36128) according to the previously reports [27]. To represent the spatio-temporal expression, a heatmap was generated (Fig. 4) on FPKM-based (Log_2_) values of the 47 *VvPOD* genes (Supplementary Table S5). Results revealed that 9 genes (*VvPOD1, VvPOD2, VvPOD6, VvPOD10, VvPOD12, VvPOD27, VvPOD32, VvPOD37*, and *VvPOD46*) displayed a striking expression levels among all tissues and organs, implicating their vital roles for grapevine. Most genes (> 15 genes,) especially *VvPOD44*, *VvPOD18*, *VvPOD4*, *VvPOD20*, *VvPOD31*, and *VvPOD38*, expressed higher in root than in other tissues, suggesting their participation in root’s developing or functioning. Moreover, the rest of the genes showed either moderate or weak expression abundance in all the selected tissues and organs, speculating their limited response in grapevine.

### qRT-PCR analysis of POD genes in response to (NaCl, drought, and ABA)

**Fig. 5:**
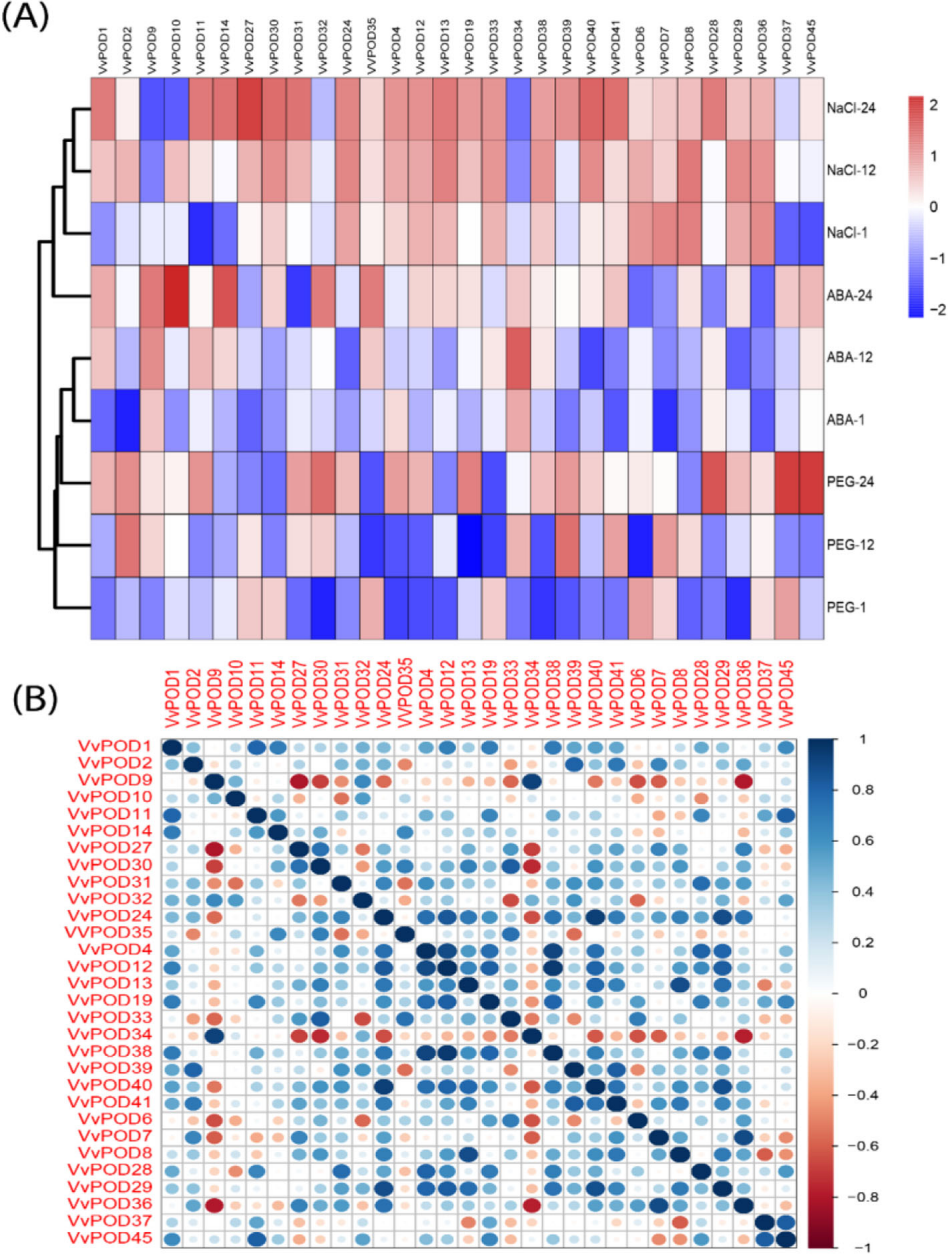
**a** Relative expression profiling by RT-PCR for significantly up and down-regulated genes under various abiotic stress (i.e., NaCl, PEG, and ABA). **b** Pearson’s correlation coefficients (PCCs) of 30 *VvPOD* genes against abiotic stress such as NaCl, PEG, and ABA and were illustrated by using RStudio

To investigate the role of *VvPOD* genes under diverse abiotic stress conditions, we performed qRT-PCR analysis of randomly selected 30 candidate genes and that were subjected to NaCl, drought, and ABA stress treatment. The results directed that all the genes responded variably and showed higher, moderate or low expression level compared to the controls. In response to salt stress, approximately 52% of the total genes showed higher expression level, whereas the rest of the genes showed either moderate or low expression. Interestingly, in the case of ABA and drought stress, about 78 and 72% genes were observed to be down-regulated (Fig. 5a and Supplementary Table S6). Most of the genes decreased their expression at the early stress periods (1 h and 12 h), but they tended to increase their expression afterwards (24 h). The expression of seven genes (*VvPOD8*, *VvPOD12*, *VvPOD19*, *VvPOD24*, *VvPOD29*, *VvPOD38*, *VvPOD39*, and *VvPOD40*) was increased 24 h after the treatment under all the stress conditions at; whereas only the transcripts of *VvPOD4034* and *VvPOD37* were decreased. Moreover, the correlation analysis based on Pearson’s Correlation Coefficient (PCC) of the relative expression indicated largely a highly positive correlation and some of them were found with inverse correlation (Fig. 5 b). Taken together, these results of *POD* genes based on expression level respond to multiple stresses and might play an important role in the maintenance of plant growth.

## Discussion

The PODs multi-gene family are involved in the various biological process by regulating plant growth and developmental processes. While, POD family members have been comprehensively analyzed by genome-wide approaches in several species including, *Arabidopsis thaliana* [20], *Oryza sativa* [21], *Panicum virgatum* [28], *Populus trichocarpa* [22], *Medicago sativa* [23], *Zea mays* [24]*, Pyrus bretschneideri* [25]. However, to date, no previous bioinformatics analysis have been carried out in grapevine for this important gene family. Also, the available genomic resources for grapevine (http://genomes.cribi.unipd.it/grape/) provides useful information and tools for the analysis of *POD* gene family in grapevine. In this study, a total of 47 *POD* genes were identified in grapevine and is known to be the largest gene families in woody plants [25]. We comprehensively analyzed physicochemical properties, phylogenetic relationships, chromosomal mapping, gene collinearity analysis, motif composition and gene structure organization, and evolutionary analysis for the duplicated pairs of POD. GO, KEGG, cis-regulatory elements, expression profiling of spatio-temporal response, and qRT-PCR analysis in response to (NaCl, drought, and ABA) disclosed extensive information on the gene functions and expression dynamics of tissue-specific and abiotic stress response in grapevine.

We determined the phylogenetic relationships between grapevine (*VvPOD*) and (*AtPOD*) by comparative analysis. Results revealed an identical domain composition of *VvPOD* with the model plant. The phylogenetic tree was categorized into 7 subgroups and the result of our tree are consistent with previously reported study of *PODs* in Cassava [29]. The motif composition analysis also demonstrated that motifs 1–4 are common among all the POD members with highly conserved nature. Moreover, the comparative structure analysis of POD showed that same subgroup shared a common junction. These results indicated a possible structural diversification within *VvPOD* gene family, which plays an important role during the evolution of multi-gene family [30].

Gene duplications are the vital force in the process of genomic evolution and functional divergence [31]. Importantly, the gene duplication is considered a major component in the establishment of new genetic functions and evolutionary novelty [32, 33]. Similarly, in the process of evolutionary history, most of the higher plant underwent polyploidization that is vital ingredient in shaping plant genome [34]. In this study, the types of duplications in grapevine were identified by the help of MCScanX among *POD* genes. Results showed 4 types of duplications including, dispersed (20), tandem (15), segmental or whole-genome duplication (9), and proximal (3). It is noteworthy that during the process of evolution segmental and tandem duplication plays a critical role in the expansion of gene family [25, 35, 36]. Though, in this study, we determined that both dispersed and tandem duplications contributed mainly to the expansion of *POD* gene family in grapevine. The selection pressure analysis are mainly based on three selection such as purifying (*Ka/Ks* < 1), positive (*Ka/Ks* > 1), and neutral selection (*Ka/Ks* = 1), and its evolutionary assessment provide useful guidelines during the rate of divergence [37]. Results inferred that 15 pairs of POD have shown less than 1.00 *Ka/Ks* ratio, indicating purifying selection and 7 pairs exhibiting more than 1.00 suggested the positive selection. The results of our study are also in consistent with previous reported studies on PODs [35, 38].

In this study, we utilized the publically available RNA-seq data of 19 different tissues and organs to validate the tissue-specific response of 47 *VvPODs*. Among them, 9 genes (*VvPOD1, VvPOD2, VvPOD6, VvPOD10, VvPOD12, VvPOD27, VvPOD32, VvPOD37,* and *VvPOD46*) exhibited significantly higher expression level among all the tissue and organs. The rest of the other genes showed down-regulation with similar tendency throughout grapevine tissues and organs developmental phases.

To better understand the gene functions, expression profiling mainly provides a valuable clue to its regulatory role against specific treatment. Abiotic stresses considerably harbor plant growth and productivity, thus lead to economic losses. The drought severity hampered grapevine productivity and has deleterious impacts on viticulture worldwide [39, 40]. Salt stress is one of the severe abiotic stress, while grapevine plants generally modify their physiology to combat salt stress severity [41]. In regulating plant tolerance to several abiotic stress, an eminent hormone, such as ABA plays an essential role. Many researchers have indicated that plant peroxidases are involved in various cellular processes during plant growth and development, and its response to abiotic and biotic stress have been reported over the years [24, 42]. For instance, membrane-bounded peroxidase (*pmPOX1*, *pmPOX2a*, *pmPOX2b,* and *pmPOX3*) in *Zea mays* roots are regulated by methyl jasmonate, salicylic acid and pathogen elicitors [19]. Similarly, the overexpression of *CpPrx01* altered the growth pattern of plants and condensed the level of a well-known hormone, indole-3-acetic acid (IAA) [43]. Thus, in this study, the qRT-PCR validation of 30 *POD* genes in response to NaCl, drought, and ABA displayed a range of differential expression. The salt stress increased the expression of most *POD*s, while drought and ABA stress decreased their expression. This suggested their important roles in grapevine against abiotic stresses. Taken together, the POD genes analysis based on RNA-seq and qRT-PCR supporting the hypothesis that these genes plays a vital roles in various environmental stimuli and development of grapevine. Hence, these analysis provided a basic resources for the examination of grapevine development and stress resistance.

To improve and comprehend the possible transcriptional regulation functions of PODs, we also analyzed the GO, KEGG enrichment, and cis-elements in grapevine. Thus, these results inferred their diversified functions and indirect involvement in several biological processes, such as biotic-abiotic/hormone signaling.

## Conclusions

In conclusion, we systematically identified a total of 47 *POD* genes in grapevine and were categorized into 7 subgroups, as supported by phylogenetic analysis. The GO, KEGG, and cis-elements analysis also extended our repositories on the diverse functions of PODs in plant developments during various stress-related activities in grapevine. While, the transcriptional profiling of various organs during several developmental stages and RT-PCR analysis, provide a stand-point of the PODs in improving the plant growth. Thus, the results of our study increase our understanding of *POD* genes in grapevine and laying the solid foundation for genetic improvement in other fruit crops.

## Methods

### Identification of POD genes family in grapevine

In order to identify the *POD* genes, we used the 73 reference sequences of *Arabidopsis* against grapevine (genome version 2.1) with the help of BioEdit tools. In general, we retrieved the sequences of grapevine and *Arabidopsis* from online sources such as ensembl (https://plants.ensembl.org/index.html) and TAIR (http://www.arabidopsis.org/). The domain composition was verified by using the NCBI-Conserved Domain database (https://www.ncbi.nlm.nih.gov/Structure/cdd/wrpsb.cgi) and SMART databases (http://smart.embl-heidelberg.de/) [44]. Those sequences with absent of POD domains and sequence with obvious error in length (>100aa length) were eliminated from the study before analysis.

### Phylogenetic analysis of POD gene family

For phylogenetic characterization, the multiple sequences alignment (MSA) of PODs was performed by MUSCLE [45] using MEGA 7.0 software with the default options [46]. The phylogenetic trees were constructed using the maximum likelihood (ML) method. After scrutiny of several models in MEGA 7.0, we choose the Jones, Taylor, and Thornton amino acid substitution model (JTT model) and for the reliability of resulting phylogenetic tree, the bootstrap values of 1000 replications were performed and keeping the other parameters as a default.

### Calculation of synonymous (Ks) and non-synonymous (Ka) for duplicated genes

The rate of *Ka/Ks* was carried out for various types of duplicated pairs (i.e., dispersed, proximal, tandem, and segmental) by using MEGA 7.0 [46]. The *Ks* and *Ka* ratio was intended by Nei- Gojobori method (Juke-Cantor) model in MEGA 7.0 with the bootstrap values of 1000 replicates. The various types of duplications of the *POD* gene pairs were detected by the MCScan algorithm.

### Gene structure, motifs composition, and physicochemical analysis of POD protein

For the gene structure illustration, we utilized the GFF3 file of the grapevine genome and images were implemented by TBtools software [47]. The motifs analysis of POD protein were performed by the Multiple Em of Motif Elicitation (MEME Suite) version 5.0.5 and then demonstrated by TBtools software. The following parameter was calibrated for this purpose as follows: the maximum number of motifs 10, with a minimum width of 50 and a maximum of 100 and the other parameter was set as default [48]. While, for each gene of POD, the several physicochemical properties (i.e., molecular weight (MW), isoelectronic points (PIs), and GRAVY) were intended by ExPASY PROTPARAM tools (http://web.expasy.org/protparam/). The subcellular localization was further predicted by using the WOLF PSORT online server (https://wolfpsort.hgc.jp/).

### Gene ontology (GO), Kyoto encyclopedia of genes and genomics (KEGG) and cis-elements predictions of class III peroxidase gene family

For the GO and KEGG enrichment analysis, the online panther server (http://pantherdb.org/) and the genome server was implemented (https://www.genome.jp/kegg/pathway.html) and their enriched pathways were further explored by TBtools software [47]. The POD promoter sequences (i.e., selected as 1500 bp) was initially imported in Generic File Format (GFF) from the grapevine genome. After that various cis-regulatory elements for each promoter sequence was identified by the PlantCARE database (http://bioinformatics.psb.ugent.be/webtools/plantcare/html/) [49].

### Chromosomal mapping and gene collinearity analysis of grapevine and Arabidopsis

The grapevine genomic database (CRIBI. Available online: http://genomes.cribi.unipd.it/grape/, V2.1), was utilized for the chromosomal locations of *POD* genes and were mapped based on information available. Similarly, for gene collinearity analysis, the grapevine and *Arabidopsis* relationship was verified and demonstrated with the help of Circos (TBtools software) program was applied [47].

### Plant material and methods

In the present study, we used the two-year-old potted grapevine plants (*V. vinifera* cv. Summer Black), selected from greenhouse condition (25 ± 5 °C) under 16-h light/8-h dark photoperiod and 65% relative humidity (RH) at the Nanjing Agricultural University Nanjing-China. The grapevine plants were subjected to abiotic stress including, NaCl (100 mM), drought (irrigated with 15% (w/v) polyethylene glycol), and ABA (100 μM/100 mL of water) with an interval of 1, 6, 24 h against control (CK), with three biological replicates and the three samples were mixed to make one composite sample while using the 4th unfolded leaf from cv. Summer Black grapevine. At last, all the samples were quickly frozen in liquid nitrogen and stored at − 80 °C for further use.

### RNA isolation and transcriptional profiling of POD gene family in grapevine

Total RNA was extracted from the grapevine leaves with the help of Trizol (Invitrogen) according to the manufacturer’s instructions. Then, the Primer Script RT reagent kit (TAKARA, Dalian, China) was used to convert the RNA into reverse-transcribed into cDNA. The gene-specific primers (Supplementary Table S7) was designed by the help of Becan Designer 7.9 and their specificity was confirmed using the BLAST tool against the grapevine genome. Also, we performed the RT-PCR by following the previously reported studies [50, 51], and the relative fold expression was calculated using the comparative Ct-method. The expression patterns of all *POD* gene were analyzed based on a previous study [36, 52], and for reference gene in qRT- PCR, we used the housekeeping actin gene (*AB073011*). In summary, the real-time PCR amplification reactions were performed on an ABI 7500 Real-Time PCR System (Applied Biosystems, CA, USA) using SYBR Green (Applied Biosystems, CA, USA) with three replicates. While the following PCR parameter conditions were set as follow: denaturation at 95 °C for 2 min, 40 cycles of denaturation at 95 °C for 10 s, annealing at 60 °C for 40 s, and extension at 72 °C for 15 s.

For expression profiling, we utilized the RNA-sequence data from the NCBI GEO website (https://www.ncbi.nlm.nih.gov/geo/) under the series entry GSE36128. Further, the expression levels were quantified by FPKM (fragments per kilobase of transcript per million fragments mapped), and heat maps were generated by using Rstudio (R program package) based on log_2_ FPKM values.

## Supplementary information

**Additional file 1 Figure S1.** The LOGOS of PODS were elucidated by MEME online server.

**Additional file 2 Table S1.** The basic information of *POD* genes identified in grapevine. **Table S2.** The gene ontology of *POD* gene in grapevine. **Table S3.** Kyoto Encyclopedia of Genes and Genomics (KEGG) *POD* genes in grapevine. **Table S4.** Cis-Elements of *POD* genes in grapevine. **Table S5.** The FPKM based values of *POD* genes in grapevine including, different organs, tissues, and developmental stages. **Table S6.** Relative expression patterns (log_2_) of *POD* genes in grapevine by qRT-PCR. **Table S7.** Sequences of the *POD* gene primers used for quantitative real-time PCR.

## Data Availability

All data generated or analysed during this study are included in this published article and its supplementary information files.

## Abbreviations

PODs: Peroxidase
ABA: Abscisic acid
CSD: Coding sequence
MW: Molecular weight
PIs: Isoelectric point
GRAVY: Grand average of hydropathicity
WGD: The whole genome duplication
GO: Gene Ontology Enrichment
KEGG: Kyoto Encyclopedia of Genes Genomics
MF: Molecular Functions
CC: Cellular Component
BP: Biological process
PCC: Pearson’s Correlation Coefficient
FPKM: Fragments per kilobase of transcript per million fragments mapped
