# Supplementary material for: Genome-wide identification of the class III POD gene family and their expression profiling in grapevine (Vitis vinifera L)
Source: BMC Genomics. 2020 Jun 29;21:444. doi: 10.1186/s12864-020-06828-z (PMC7325284; doi:10.1186/s12864-020-06828-z)
Supplement: Supplementary file 1 — Additional file 1 Figure S1. The LOGOS of PODS were elucidated by MEME online server. [file 12864_2020_6828_MOESM1_ESM.pdf]

	Consensus Sequences	Length (aa)
**Motif 1**	FEVIDDAKTAVESACPGVVSCADILALAARDAVVLSGGPSWKVPLGRRDGRTSSASEA	58
**Motif 2**	NLPSPTDSJSQLISKFASKGLSTKDLVALSGAHTIGFAHCSSFRSRJYNFSGTGDPDPTLNPSYAAVLQKKCPKD GDGDVALALDDSTTTNFDBBYYKNL	100
**Motif 3**	SAQLSVNFYSKSCPKAESIVRSTVRKAFIKDPRIAASLLRLHFHDCFVQGCDASVLLDSTS	61
**Motif 4**	ALFTDGRTKSIVETYAKNQALFFRDFAASMVKMGNIGVLTGTEGEIRKNC	50
**Motif 5**	EKDGLPNAGLRGFDFIEDVKSQLEAECPGIVSCADILALVARDAIGLSGGPFWNVPTGRRD	61
**Motif 6**	QGLKPGFYKNSCPKAEDIVRETVVQHFKKDPTJAAGLLKJHFKDCFFRGCDG	52
**Motif 7**	PRPVDNFPVLREKFANKGLDNNDLVLLIGAHTIGLSDCSSFENRLYNFTGKGDEDP	56
**Motif 8**	EFPKAMEKMGRIERKEGTEGEVRKFCAGGPFMEREWQVWLCVKGGLFAEK	50
**Motif 9**	KKGVPLDKGSFRKFDLSFFKNLRDGRGLFESDQRLFGDSETKRFIKNIAG	50
**Motif 10**	GVQRLIREDCFGQGCDGLFFIDCFVNECDALPNLGDRGFDVGEDNKLPNZ	50
